# Global COVID-19: Warnings and suggestions based on experience of China

**DOI:** 10.7189/jogh.10.011005

**Published:** 2020-06

**Authors:** Zhuqing Ding, Lingling Xie, Anchen Guan, Dandan Huang, Zongfu Mao, Xiaohui Liang

**Affiliations:** Global Health Institute/ School of Health Sciences, Wuhan University, Wuhan, China

## Abstract

**Background:**

Corona Virus Disease 2019 (COVID-19) is spreading around the world currently, seriously threatening people’s health and global security as an international public health emergency. The objective of this study is to summarize China’s countermeasures and experience in response to the COVID-19 emergence in order to serve as a warning for the global COVID-19 prevention and control, and further provide some suggestions for global fighting to COVID-19.

**Methods:**

Content analysis, expert consultation, and high frequency word analysis were applied in this study. Relevant data and information were collected from official websites, the experience accumulated in China during the fighting to the novel coronavirus, and suggestions from some experts.

**Results:**

As of March 29, 2020, China had accumulated 82 419 confirmed diagnoses, and the mortality rate was 4.01%; in the mean time, the global had accumulated 596 042 confirmed diagnoses, and the mortality rate was 4.76%. Although the mortality of COVID-19 was not so high, its harmfulness couldn’t be ignored. Ten facts during COVID-19 epidemic in China were summarized in the study, including: the COVID-19 is highly contagious and can be spread through many channels; although elderly people and people with underlying diseases are susceptible, young people can also be victims; isolation is the most effective way to reduce the risk of COVID-19, and secondary disasters induced by COVID-19 should be emphasized in advance. High frequent words of experts’ suggestions mainly includes: material, prevention and control, pathogeny, propaganda, education, hygiene, technology, medical care, overall planning, policy, panic, etc. The main concerns of experts are from four aspects: publicity and education, various subjects, medical materials and law construction.

**Conclusions:**

COVID-19 is a new infectious disease worldwide, and its infectious source, transmission route, susceptible population, and therapy are not clear. In the face of the COVID-19 epidemic, no country can stand alone to maintain its own safety. People from all over the world should put aside their difference in ideology, religious belief, politics, economy, and culture, and only by global cooperation and globally unified actions, can we successfully overcome COVID-19 in the end.

Corona Virus Disease 2019 (COVID-19), which is induced by novel coronavirus (2019-nCoV), was first found in Wuhan [1]. Currently, it is spreading globally. Many countries, especially those with poor health systems, are facing greater threats. When COVID-19 epidemic occurred in China, the Chinese government adopted strict prevention and control measures, which proved effective in curbing the spread of the epidemic in China [2]. However, just as the peak of the epidemic in China has recently passed, the virus transmission is becoming more severe around the world, showing the characteristics of a pandemic. Europe has become the new center of the epidemic now. As of March 28, 2020, COVID-19 has spread to 199 countries around the world, with a cumulative 462 684 confirmed diagnoses worldwide. Among them, the number of confirmed infections in America, Italy, Spain, Germany, France, Iran have already exceeded 20 000 [3]. China’s strict prevention and control has earned time for mitigation of the COVID-19. The experience and lessons accumulated in China during the exploration of the novel coronavirus deserve to be summed up, which may be useful for other countries to address the challenge. Therefore, the purpose of this study is to sort out China’s countermeasures and experience and collect some Chinese experts’ advice for fighting to COVID-19. It is expected to serve as a warning for the global COVID-19 prevention and control, and to provide a reference for the formulation of prevention and control strategies in other countries and regions.

## METHODS

### Expert consultation

A questionnaire on how to prevent and control COVID-19 was distributed to relevant experts, and their advice was collected for high frequency word analysis and content analysis.

### High frequency word analysis

The frequency of words in a particular field usually reflects its hotspots and main opinions. With regard to the feedback from the professionals and experts, high frequency word analysis was conducted using the software from the website of Tuyue (http://www.picdata.cn/picdata/).

### Content analysis

Both experts’ feedbacks and relevant information about China’s countermeasures collected from official websites were analyzed and summarized. In addition, the study also sorted out the experience and lessons accumulated in China during the fighting with the novel coronavirus.

### Statistic analysis

All data about the new and cumulative cases of China and the global were crawled from Toutiao of China (http://www.toutiao.com). It is an open database of COVID-19 collected from national and provincial Health and Family Planning Commission. The data were performed by SPSS 24 via descriptive-analytical statistics.

## RESULTS

As of March 29, 2020, China had accumulated 82 419 confirmed diagnoses, and the mortality rate was 4.01%; in the mean time, the global had accumulated 596 042 confirmed diagnoses, and the mortality rate was 4.76%. Based on the statistics and relevant information, China’s countermeasures and some facts for the COVID-19 epidemic in China were summarized. There were total twelve experts taking part in this survey, and they were from various organizations, including hospital, government agency, research institute and university, etc. The results of high frequent word analysis and content analysis were used for providing references for suggestions.

### Chinese countermeasures to COVID-19

Since the outbreak of COVID-19, under the leadership of the government, Chinese people have united and worked together to provide special support to Wuhan, the city with the worst epidemic. The following is a short review of the process of epidemic prevention in terms of transmission control, policy support and scientific research.

#### Transmission control

Restricted traffic and cities: On January 14, 2020, the local government of Wuhan implemented the control of personnel departure, and set up temperature detection points and investigation points at airports, railway stations, coach stations and passenger terminals [4]. On January 22, Hubei province launched a secondary public health emergency alert. Wuhan closed the city on January 23 [5], and from January 23 to January 29, 31 provinces in China successively initiated first-level responses to major public health emergencies [6]. Many regions practiced closed-off management then. From March 4, 21 provinces, autonomous regions, and municipalities nationwide lowered their emergency response levels [7].

Closed zone management: Closed management was implemented in urban communities or rural villages. Non-community or non-village residents or vehicles were not allowed to enter the communities or villages; even the local residents needed to have their temperature measured when they were allowed in and out of the area. In the community or village areas, the epidemic prevention and control groups were established. These could be divided into many subgroups with different functions, such as: propaganda group, environment group, key personnel prevention and control group, personal in and out control group, material support group others, in order to complete the epidemic prevention and control work in an all-encompassing manner [8].

Personal management: From February 16, citizens in Wuhan were required to scan and enter identifying information when they assessed any open public places [9]. On March 5, 2020, “the reference architecture and technical guide of epidemic prevention access code” was issued. According to this regulation, Chinese citizens had to report their health status online and had their general health status captured by two-dimensional codes. The code could also work as an electronic voucher for citizens to go in and out of the local places. The health assessment based on two-dimensional codes was universal in the whole city, even the whole country [10].

#### Policy support

Staff support: On January 23, Shanghai and Guangdong sent the first medical teams to Wuhan [11]. On January 28, 6097 people across 52 medical teams nationwide went to support Hubei [12]. As of March 8, a total of 346 medical teams and 42 600 medical staff arrived in Hubei, fighting the virus alongside with local medical staff [13].

Funding support: On January 26, National Development and Reform Commission urgently issued 300 million Yuan (RMB) to support Hubei from the central budget, and 11.21 billion Yuan was issued from national finances at all levels in subsidies for disease prevention and control [14]. As of March 4, 110.48 billion Yuan had been arranged from the government’s finances at all levels for prevention and control [15].

Facility construction: Wuhan was the city with the worst epidemic situation, and it has become the key area for the epidemic prevention and control. In order to fight COVID-19, Wuhan set up “Vulcan Mountain” and “Thor Mountain” Hospitals, which only took about 10 days to finish, to treat severe patients [16]. In addition, 11 Cabin Hospitals were built for mild patients, and a dozen of hotels and schools were recruited to isolate mild patients and close contacts [17]. The policy for the patients, as well as the close contacts, was changed from self-isolation at home at the beginning to all-isolation at hospital or hotel at the end.

#### Scientific research

Recognition of novel coronavirus: The understanding of novel coronavirus was a gradually developing process. On December 8, 2019, Wuhan Municipal Health Commission notified the first confirmed case that showed symptoms on that same day. On January 2, 2020, the whole genome sequence of novel coronavirus was obtained by the Wuhan Institute of Virology, Chinese Academy of Sciences [18]. At a press conference of the National Health Commission of the People’s Republic of China on January 20, Professor Zhong Nanshan confirmed that the novel coronavirus was transmitted from person to person [19].

Detection reagents and treatment plan: In terms of detection reagents, on January 4, China’s Centre for Disease Control developed a specific PCR detection reagent. On January 11, China’s Centre for Disease Control provided PCR detection reagents to Wuhan hospitals. On January 26, the National Medical Products Administration approved 4 novel coronavirus detection products from 4 Chinese companies [20]. Regarding the diagnosis and treatment plan, on January 15, the National Health Commission of China issued the first Trail Edition of Diagnosis and Treatment Plan for COVID-19. By February 19, the versions were constantly updated. The seventh versions of the diagnosis and treatment plan had been issued [21]. The diagnostic criteria changed from the diagnosis based on the results of the novel coronavirus PCR test to the diagnosis based on clinical CT results.

Drug research and development: On January 28, the research and development of novel coronavirus mRNA vaccine was officially initiated [22]. On February 5, the clinical trials of Remdesivir began [23]. On February 21, the Ministry of Science and Technology stated that the vaccine should be submitted for clinical trials as soon as late April [24]. On March 6, Chloroquine Phosphate became a clinical treatment medication [25]. In addition, some Chinese traditional medicines have been demonstrated to be effective [26].

### Results of China’s countermeasures

**Figure 1:**
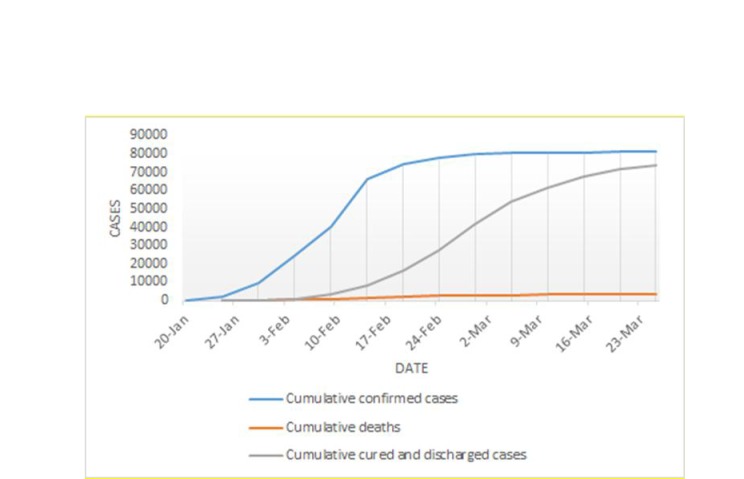
Trends of cumulative confirmed, dead, cured and discharged cases in China.

**Figure 2:**
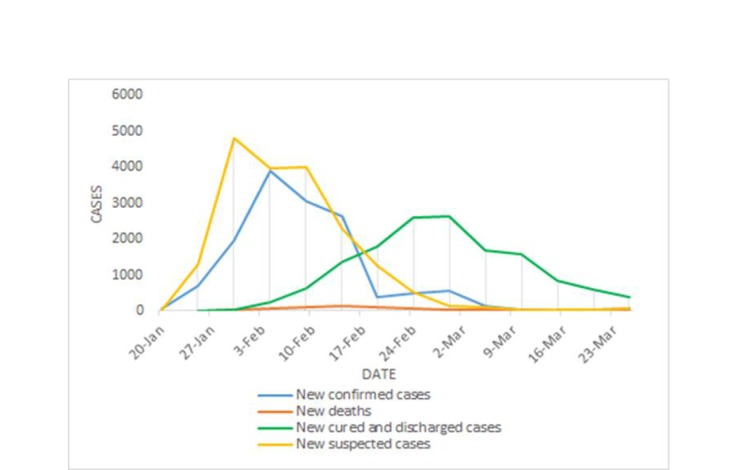
Trends of new confirmed, dead, suspected, cured and discharged cases in China.

Facing the severe situation brought by COVID-19, China’s government has adopted a series of strict prevention and control countermeasures, which have effectively controlled the spread of the epidemic in China and have gained time for the prevention and control of COVID-19 for the other countries. According to the daily outbreak data released by the National Health Commission of China, from the national point of view, the mortality rate of patients has been declining, and the proportion of patients with severe illness has been decreasing. The trend of cumulative cases in China is shown in **Figure 1** and the trend of new cases in China is shown in **Figure 2**.

**Figure 1** and **Figure 2** shows the changes in relevant national data since the outbreak of COVID-19. The numbers of newly diagnosed patients, suspected patients, as well as newly added deaths have risen for a period of time and are currently in a declining phase. Current data demonstrates that China’s countermeasures are effectively slowing down the spread of COVID-19. It may give a piece of evidence or experience for global COVID-19 prevention and control in the near future.

As of March 29, China has accumulated 82 419 confirmed diagnoses, 3506 existing confirmed diagnoses, 3306 deaths, and 75 600 cured patients [27]. Based on the current data, China’s cure rate is 91.73% and the mortality rate is 4.01%. It is worth noting that there were 693 cases that were imported from overseas. **Table 1** shows the Wuhan, Hubei, national and global cumulative number and mortality rate. The data demonstrates that the mortality rate could obviously drop down with sufficient medical resource, for example: the mortality rate in the rest of China, excluding Hubei, is much lower that other places. It is quite clear that the overall case-fatality rate is based on the denominator that includes those who were positively tested and not all those infected with symptoms.

**Table 1:** Comparison of the number of cumulative COVID-19 cases and mortality among Wuhan, Hubei, China, and the world

Regions	Total number			Mortality rate (%)
**Cumulative diagnosis**	**Cumulative death**	**Cumulative cured**
Wuhan	50 006	2543	45 418	5.09%
Hubei (excluding Wuhan)	17 795	639	17 147	3.59%
China (excluding Hubei)	14 618	124	13 042	0.85%
China	82 419	3306	75 607	4.01%
Global (excluding China)	596 042	28 342	65 600	4.76%
Global (including China)	678 461	31 648	141 207	4.66%

### Ten facts about COVID-19

Considering the case-fatality rate of COVID-19 in China (4.01%) and the globe (4.66%), some people may consider this disease to be only affecting those with underlying diseases and mainly old persons. However, based on China’s experience in treating those with pneumonia, this would not be entirely true. Ten facts of China’s novel coronavirus pneumonia are listed here. These ten facts are well worth global vigilance.

#### Many medical workers were infected in hospitals

According to investigations, more than 3000 medical staffs in Wuhan have been infected since the outbreak [28]. It is a warning to the world that the infection rate of the novel coronavirus is very high. More attention must be paid to the infection of medical staff and nosocomial infection.

#### Home isolation leads to increased household cluster infections

When China adopted the “home quarantine” policy at beginning, there was a large number of household cluster infections, and even tragedies where there would be deaths of entire families [29]. This proved again the high infection rate of the novel coronavirus and that “home quarantine” policy may be not a good choice. Once infected by COVID-19, patients are better isolated and separated from their families.

#### Young people can also be victims

Although it is generally believed that the elderly and patients with underlying diseases have higher mortality rates [30], it has been proven that healthy young people also can develop severe illness. The severity of the disease may be related to cytokine storm^[^31^]^. In addition, there were still a few cases of infant or children infection.

#### The results of molecular diagnosis may be both false negative and false positive

At beginning, the clinical diagnosis was based on detecting method of molecular biology – PCR (polymerase chain reaction). When throat swabs or nose swabs were taken as sampling, there were some patients whose test results showed negative. One of the patients was false negative 5 times [32]. Meanwhile there were also “asymptomatic carriers” with positive molecular diagnosis results, but no symptoms [33]. Because of false positive and false-negative results, molecular diagnosis, being the unique diagnostic criteria, is challenged. Later, combining PCR with clinical CT results was recommended as the basis for the diagnosis of COVID-19.

#### The patient’s condition might suddenly rapid worsen even during the recovery period

During treatment, there were some clinical cases in which convalescent patients had significantly improved symptoms. However, the patiens’ condition would suddenly deteriorate and they would pass away within only a few hours. The mechanism of death cause remains unknown.

#### COVID-19 causes serious shortage of medical supplies

On the one hand, COVID-19 outbreaks can cause serious medical supplies shortage. On the other hand, the public’s panic can make it much worse. The shortage of medical materials, staff and supplies should all be expected. This phenomenon deserves attention in other regions of the world. The storage of medical materials is particularly important, especially in the early stage of the outbreak.

#### Secondary disasters were caused by COVID-19

Due to the large amount of medical resources invested in the treatment of the novel coronavirus disease, other clinic treatments, such as surgery, emergency medical treatment, tumor radiotherapy and chemotherapy were not available [34]. This lead to increased mortality in patients with other diseases in the hospital.

#### There is no specific and effective treatment for COVID-19

The clinical treatment for COVID-19 is mainly oxygen inhalation and symptomatic treatment. There is no specific and effective drugs for saving life of severe patients at present [35].

#### Multi participation and unified action are the guarantee of epidemic control

Since COVID-19 first occurred in Wuhan, both the central government and the local government in China had paid great attention to it. Besides, more than 40 000 medical staff from other provinces were sent to Wuhan. Other participants from transportation, traffic and military industries actively took part in preparing medical material and food supplies. It took a national-level unified action to effectively control the COVID-19.

#### Isolation is the most effective way to reduce the risk of COVID-19

Strict closure of cities and quarantine measures have demonstrated that Chinese isolation counter-measures have achieved good results. The achievement is that Wuhan will be released from closure in April 8. Isolation measures can effectively reduce the COVID-19 incidence rate and save many people’s lives. Therefore, the isolation counter-measure is worth being recommended to other countries’ policy makers.

### Experts’ advice for COVID-19 control in China

Twelve Chinese public health professionals or Disease Control Center experts were investigated about how to prevent and control COVID-19 recently. Their advice was analyzed by high frequency word analysis and content analysis.

#### Results of high frequency word analysis

Keywords are used to generalize the literature’s content. Keyword frequency may reflect the main content of a group of the documents. **Table 2** shows the top 30 keywords with their frequency and weight value. Integrating them with the high frequency terms, we found that experts’ advice for preventing and controlling COVID-19 included these specific keywords: material, prevention and control, pathogeny, propaganda, education, hygiene, technology, medical care, overall planning, policy and panic. These keywords reflect some main concerns that Chinese experts had about COVID-19.

**Table 2:** Top 30 keywords with high frequency and weight value from experts’ advice

Top 1-15	Keywords	Frequency	Weight value	Top 16-30	Keywords	Frequency	Weight value
1	material	19	1	16	disease	6	0.8304
2	prevention & control	15	0.9934	17	technology	7	0.8296
3	epidemic situation	15	0.9826	18	Public Health	5	0.8288
4	pneumonia	8	0.8988	19	Healthy	6	0.8203
5	mechanism	10	0.8869	20	Medical care	6	0.818
6	distribution	10	0.8849	21	quarantine	5	0.8166
7	protect	8	0.8799	22	The basic level	5	0.8146
8	guarantee	9	0.8772	23	Coronal	4	0.8129
9	Train	9	0.8758	24	countryside	5	0.8091
10	Pathogeny	7	0.8581	25	Overall planning	5	0.805
11	virus	7	0.8528	26	knowledge	5	0.8038
12	Infectious Diseases	6	0.8483	27	Infected	5	0.8031
13	Propaganda	7	0.8461	28	spread	5	0.8028
14	education	7	0.8421	29	policy	5	0.8004
15	Hygiene	6	0.8315	30	panic	4	0.7943

#### Results of content analysis

Content analysis was used to summarize experts’ advice for preventing and controlling COVID-19. The main advices were sorted and analyzed from four aspects: publicity and education, various subjects, medical materials and law construction. Chinese experts’ advice is listed in **Table 3**.

**Table 3:** Chinese experts’ advice for preventing and controlling COVID-19

Aspects	Main advice
**Publicity and education**	To carry out various forms of publicity, including Internet publicity, TV programs, newspapers, books, comics, lectures, etc., so that people can fully understand the new crown pneumonia and how to effectively protect them
	To reducing social panic by unified voice of authoritative media
**Various subjects**	For ordinary people: to strictly control the flow of personnel
	For COVID-19 Patients: to be admitted to hospitals
	For asymptomatic and close contacts: to be isolated from home isolation mode to centralized isolation mode
	For non-COVID-19 other patients: to guarantee normal basic treatment decreasing secondary disasters
	For front-line medical staffs: to be trained before they are on duty, and be provided sufficient protection materials, salary and remuneration
	For the grass-roots staffs: to open the community prevention and control mechanism and strengthen the basic protective measures
	For scientific research: to strengthen international cooperation and scientific research, and strive for new progress in drug research, vaccine development and epidemiological investigation.
**Medical materials**	To meet the medical material needs of the hospitals for prevention and treatment of COVID-19
	To meet the ordinary people’s needs of prevention and control materials and basic daily necessities
	To guarantee the production, circulation and reserve of all medical materials for COVID-19
**Policy and law construction**	To severely punish those persons who disturb the stability of the market, such as driving up prices and hoarding
	To severely punish these persons who make rumor and fraud that cause public panic
	To severely punish the behavior of disobeying the management and escaping from the isolation area without permission
	To respect and protect the personal information, rights and interests of the COVID-19 affected population, and avoid privacy disclosure
	To avoid COVID-19 inducing district stigmatization and discrimination
	To fully take advantage of information and communications technology (ICT) in disease control, diagnosis and treatment
	To establish a long-term mechanism for infectious disease monitoring and public opinion surveillance
	To establishing a long-term mechanism for training public health global health and emergency professionals.

## SUGGESTIONS

The epidemic prevention of COVID-19 is not only a battle fought by Wuhan, but also a battle of China and eventually the world. Based on analyzing the experience and lessons of China’s epidemic prevention and control work, and consulting the opinions and suggestions of some Chinese experts, we propose the following advice for the global COVID-19 prevention and control:

### To launch a health education campaign to correctly understand COVID-19

First of all, in addition to making full use of the two important communication tools, television and the internet, various forms of publicity should be used to make the public fully aware of the severity of COVID-19. The public should be well informed of the novel coronavirus. In this special times, it is emphasized to not attend large gatherings, wash hands frequently, cover mouth and nose when sneezing or coughing, wear a mask if necessary and strengthen awareness of prevention. Second, it is important to report the epidemic information in a timely and accurate manner. This can effectively avoid panic and mistakes due to misinformation.

### To cut off the route of transmission using isolation

COVID-19 is highly infectious. Except for patients with obvious symptoms, mild or atypical patients and even asymptomatic infections are all possible sources of transmission. Therefore, controlling the source of infection and cutting off transmission routes are fundamental measures for prevention and control of the epidemic. The Wuhan closure, work stoppage, school suspension and community closed management, as well as the first-level response to major public health events launched nationwide, are measures that effectively controlled the movement of people and the spread of the epidemic. Isolation as an effective approach is worth promoting around the world. On the other hand, home isolation may lead to household cluster infection, which is noteworthy. Therefore, if possible, as to all COVID-19 patients, they should be treated in COVID-19 designated hospitals with a centralized treatment plan to reduce nosocomial infection. As to all susceptible persons or close contacts, they should be asked to stay in hotels, idle factories, large sports grounds or standby isolation places for centralized isolation. All infected patients and close contacts can be found by self-conscious online reporting. Currently, isolation may be the unique effective way to cut off the route of transmission and decrease the incidence of COVID-19. In addition, in order to prevent the spread of infection caused by secondary pollution, more attention should be paid to a reasonable disposal of COVID-19 medical waste.

### To protect the susceptible groups, especially the high-risk groups

According to a survey from Hubei, 40% of COVID-19 infections are in hospitals and 60% in communities. This is a warning for global epidemic prevention. The susceptible groups of COVID-19 include front-line medical staff, elderly population, and the gregarious groups (including nursing homes, residential schools, barracks, prisons, etc.). Therefore, the protection provided for them must be given priority in the global work of prevention and control.

### To take advantage of internet technology and reasonably allocate medical resources

In the response to the outbreak, combination of online and offline medical consulting in China have been effective to solve the problem of shortage of clinical doctors. In the global work of prevention and control, relevant countries and regions should allocate medical staff reasonably, triage patients properly before or during admission, improve the efficiency of treatment and prevent cross-infection. Adequate medical supplies and orderly distribution are important to effective epidemic prevention and control. Sufficient human and material support are helpful to ensure the success of treatment of non-COVID-19 patients.

### To fight against COVID-19 by global cooperation and globally unified actions

No country around the world can deal with global epidemic on its own. Health is a global issue, and global health is local. Thus, the fight against COVID-19 relies on local efforts in every country around the world. It is important for all countries to timely share the indicators and information with the WHO. The global cooperation should include three aspects. First, the world should join hands together to share information of the epidemic situation openly and transparently. Second, scientists in the world should cooperate to study the origin of the virus, formulate diagnostic criteria, search for specific clinical drugs, develop vaccines and jointly fight against COVID-19. Third, all countries should have unified action under the leading of WHO. Under the global unified action plan, the duration for fighting the COVID-19 will be shorter.

## CONCLUSIONS

In the era of globalization, all nations share weal and woe. In the face of the COVID-19 epidemic, no country can stand alone and only maintain its own safety. People from all over the world should put aside their differences in ideology, religious belief, politics, economy and culture. All countries should work together to fight against the virus instead of shifting blame and suspecting each other, It must be condemned for any government or individual to take advantage of the new pneumonia to stigmatize some other countries. Mankind is a community of destiny. Only by closer cooperation and mutual help with all countries, can we successfully overcome the pandemic in the global war against COVID-19.
